# The ‘experimental public’ in longitudinal health research: views of local leaders and service providers in rural South Africa

**DOI:** 10.1186/s41256-017-0046-7

**Published:** 2017-09-06

**Authors:** Rhian Twine, Gillian Lewando Hundt, Kathleen Kahn

**Affiliations:** 1 MRC/Wits Rural Public Health and Health Transitions Research Unit (Agincourt), School of Public Health, Faculty of Health Sciences, University of the Witwatersrand, Johannesburg, South Africa; 2 Division of Health Sciences, Warwick Medical School, University of Warwick, Coventry, UK; 3 INDEPTH Network, Accra, Ghana; 4 Epidemiology and Global Health Unit, Department of Public Health and Clinical Medicine, Umeå University, Umeå, Sweden

**Keywords:** Experimental public, Longitudinal health research, HDSS, Rural, Ethics of practice

## Abstract

**Background:**

The concept of ‘experimental public’ has been recently applied to publics involved in clinical trials. This term could also be applied to publics involved in longitudinal research such as health and demographic surveillance systems. The ethics of practice and public engagement with these experimental publics are of key importance and include issues of informed consent, confidentiality, collection of body tissue samples and fair local benefit.

**Methods:**

Individual (*n* = 11) and focus group (*n* = 5) qualitative semi-structured interviews were conducted with 56 local leaders and service providers regarding their views about research activities in a longitudinal health research study site run by the MRC/Wits Rural Public Health and Health Transitions Research Unit (Agincourt) in rural South Africa. Deductive and inductive thematic analysis was undertaken using NVivo software to identify the emergent themes.

**Results:**

There was an understanding of the usefulness of collecting demographic data, but reasons for gathering other contextual data such as on food security, as well as the reasons for collection of blood was less clear. While appreciation was expressed for feedback of individual results such as blood pressure levels during home-based data collection, there were requests for more results from biomarkers, and for these to be given at home, rather than at the clinic. There were reports of indirect refusals, and offers by leaders to assist in reducing refusal rates. There were concerns about confidentiality, especially in the publication of results. Some leaders would have liked to receive more individual level data for planning of services, although they understood this would breach confidentiality. Service providers were concerned about the withdrawal of some services post intervention trials.

**Conclusions:**

This experimental public has, over time, developed a nuanced understanding of the reasons for research and the procedures undertaken. Discussions concerning fair benefit ranged from requests for more individual clinically-relevant results for participants, to understanding how research results could assist in planning of public health services at local and national levels. The concerns illustrate the complexity of the ethics of practice which has implications for policy, practice and governance for those working in longitudinal health research sites globally.

## Background

There has been some recent discussion of publics in public health in Africa [1] which critically explores the complexities, dimensions and dynamics of working with and being part of publics in global health research in Africa. This paper is a contribution to the emergent literature in this field through an analysis of the views of local leaders and service providers about the activities of a longitudinal health research site which operates in rural South Africa.

The concept of ‘experimental publics’ has been proposed by Montgomery and Pool (2017), rather than ‘trial communities’ in relation to study participants of clinical trials. Their rationale is that ‘communities’ is a term that is employed uncritically when in reality the individuals or clusters involved in trials or public health research are socially constructed with geographic, demographic or health-related inclusion and exclusion criteria. This paper extends the concept of experimental publics by applying it beyond a time-bound clinical trial, to a population being studied within a longitudinal health and socio-demographic surveillance system (HDSS). Through prospective census rounds, an HDSS monitors health, social and demographic variables of an entire geographically defined population. Special modules and status observations can be added to provide more detail, such as education, food security, health care utilisation and labour migration [2]. The MRC/Wits Rural Public Health and Health Transitions Research Unit (Agincourt) has run an HDSS since 1992. It is within this research setting that the data for this paper were collected. Scientific papers covering close to 25 years of research, have generally referred to the population living in this research site as ‘the community’ [3–9]. All households in the original study villages have been included in an annual census since 1992 and in newer villages as they were added to the site. Data has been collected on all individuals, and as such all residents, both permanent and those who temporarily migrate out for work, study and other purposes, form an experimental public. Within this overarching experimental public are nested research studies covering diverse study designs, which sample participants according to specific inclusion criteria; these participants form smaller, time-bound experimental publics.

Another emergent area is the ethics of practice in public health research and the increasing attention and debate about the processes, relevance and benefit of health research to the population living in low and middle income countries [10, 11] and specifically in HDSS sites [12, 13]. Public perceptions and expectations in longitudinal research studies need to be taken into account [14], and there is an increased onus on public health researchers to develop public engagement strategies that aim to bring local value to the research itself [15–18].

A distinction has been made between *procedural ethics* (theory and regulatory board requirements) *and ethics in practice* (dealing with ad hoc situations occurring during field research) [19], which can also extend to include a socio-political approach to the ethics of practice drawing on relevant sociological and anthropological approaches [20]. Areas of ethics of practice that will be dealt with in this paper are informed consent [18, 21], confidentiality and anonymity [22–24], taking blood samples for screening [25], providing participants with blood results, and fair benefit when studies end [26, 27].

There is a body of literature exploring the complexities of the consenting process in rural African contexts [28, 29]. The consenting process is often influenced by cultural, gender and social norms, and this can affect the ‘voluntariness’ of participants’ decisions [18, 21]. In longitudinal surveillance sites, where the entire population is the experimental public, consent is often negotiated collectively (as in village or household) as well as individually [30]. This approach may affect the potential participants’ freedom to directly refuse to participate in studies, and result in actions such as not honouring appointments after consenting to participate. Kamuya et al. [31], in a longitudinal health research site in rural Kenya, describe this ‘hesitating to participate without explicitly refusing’ as a ‘silent refusal’ (3:2015). Their work illustrates that although ethical principles such as autonomy are universally applied [26], such principles need to be negotiated in different cultural contexts [28, 31].

Ensuring confidentiality and anonymity are part of both procedural ethics and ethics in practice. In HDSS sites the location of the experimental publics is described, often with maps, in many publications. While the participation in research studies may have individual or collective health care benefits, there is a possibility that research results (such as HIV prevalence or cause-specific mortality rates) may be construed by others to typify only the experimental public rather than the general population [22, 32]. An additional concern is that local fieldworkers are often employed to conduct interviews, since they speak the local language, and share the local culture. However, study participants might not trust them to keep confidentiality [24].

Concerns about body tissue collection have been raised by experimental publics for many years as ways to express deeper issues about the configuration of international health research in Africa. In HDSS sites, there may be trials or observational studies that include the collection of blood and other tissue samples. Rumours around blood taking are built through historical experiences and social belief structures, influenced by cultural practices [33–36]. In one instance there were rumours that blood was being sold for cash by the researchers in the context of a microbicide gel trial in South Africa, in which reimbursements were given for participation in the trial [25]. Clinical researchers and service providers involved with the trial saw these stories as exemplifying misunderstandings, but the authors interpreted these lay explanations as a critique of relations between researchers and local participants, expressions of popular resistance and related to local ideas of gender and morality.

Another important issue with specific reference to HDSS and other longitudinal research sites is the sustainability of services provided to study participants forming the experimental public when health service intervention studies end [30]. There has been more literature on obligations of researchers in relation to post clinical trial settings [37–39] than about public health service interventions [10, 26]. These ethics of practice issues are explored in this paper through an analysis of the views of local leaders and service providers on the past and present research activities in one site. The aim of this paper is to contribute to the emerging debate on the ethics of practice, and focusses on the understandings of local leaders and service providers, as part of the experimental public in a longitudinal health surveillance study site, concerning the research activities.

## Methods

### Setting

**Fig. 1:**
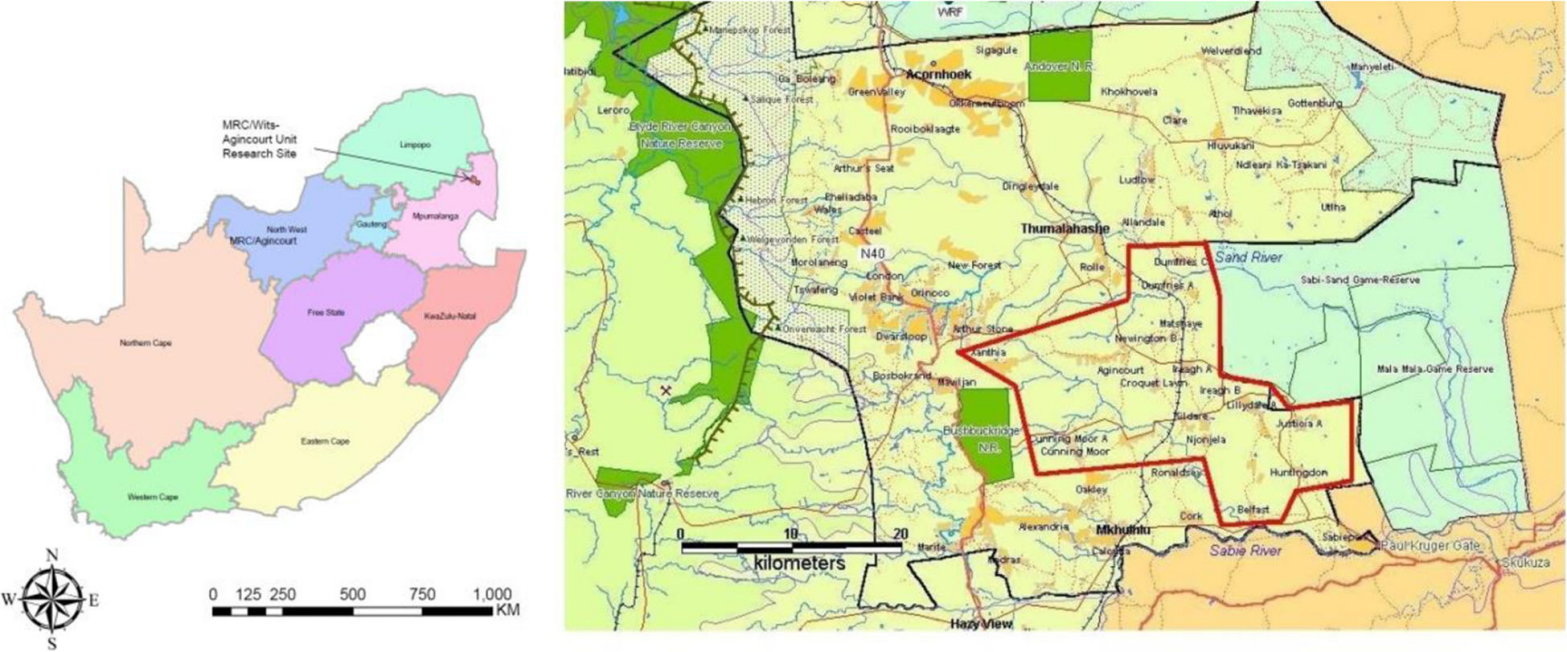
Agincourt HDSS study area in South Africa and details of the HDSS site in 2007. Source: adapted from Freeman, P.H 2002 [54]

This work was undertaken within the MRC/Wits Rural Public Health and Health Transitions Research Unit (Agincourt) research site that hosts a longitudinal HDSS established in 1992. The research site is based in the rural Bushbuckridge Municipal sub-district in Mpumalanga Province, northeast South Africa, 500 kms from Johannesburg (Fig. 1). The Bushbuckridge sub-district was part of the Gazankulu ‘homeland’ during the pre-1994 apartheid era. Some 30% of the sub-district population comprises former Mozambican refugees, owing to the area’s location alongside the western border of Mozambique.

The research site was expanded in 2007 and again in 2014 to accommodate trials and now includes 27 adjacent villages [6].

A ‘village’ is defined as a cluster of households in a geographically defined area, which has a name and recognised leadership structure, and is geographically separate from other villages. These villages fall under three Traditional Councils, and three Municipal Offices. The power and influence of the traditional leaders (indunas) and their traditional councils has decreased considerably since democratic change in 1994, but they are still respected leaders in the community and are consulted on most matters. Local political governance operates at three levels – Municipal, Ward and Community Development Forum (CDF). There are six municipal wards, each of which has a ward councilor who is accountable to the municipal offices and to the CDF. Each village CDF is made up of two representatives from every Community Based Organisation in the village, as well as the Induna (Chief) as a representative of the Traditional Council.

Since 1994, there has been some development of the region’s infrastructure evident in tarred roads, prepaid electricity, and improving yet inadequate water provision. Quality of education remains poor although every village has at least one primary school and most a high school. In the pre-2008 study site, where participants for this study were drawn, there are two health centres and six clinics, with three district hospitals 25–60kms away [3]. Unemployment is high with most formal employment being male men migrating to work in industry, largely mining, manufacturing and agriculture, but with an increasing number of women joining the temporary migrant labour force. Remittances from these migrant labourers, as well as South Africa’s non-contributory social grant system are the main sources of household income [40].

The Agincourt HDSS, which updates vital events annually, including births, deaths, and migration in and out , was established in 1992. Since 2000, in addition to the annual census update, there has been a growing number of observational, intervention and evaluation studies with international and local collaborators. Public engagement has been ongoing and in 2004, a dedicated Public Engagement Office (PEO) was established to further develop knowledge brokerage activities through public engagement with the experimental public within the site, i.e. villagers, local representatives and service providers. This office engages locally regarding forthcoming research activities and organizes village-based dissemination of HDSS results and research findings. Annual meetings are held with local service providers to discuss the relevance of research results for their work. A Community Advisory Group consisting of nominated representatives from each village meets monthly and all research projects are discussed. All research project leaders have to work with the PEO concerning how to conduct community entry for each project, and the PEO delivers training on and monitors informed consent processes and facilitates dissemination of research results.

### Research design

This is a qualitative study using semi-structured individual and focus groups interviews carried out in 2015–16. RT conducted the interviews with the help of a fieldworker acting as note taker, except for the interviews with the indunas where their roles were reversed. A purposive sample was selected of individuals working within organisations involved in governance or service provision at the village and sub-district level. Since this paper focuses on longitudinal engagement in research, we have included only the 19 villages which have been within the study site from its inception in 1992 and four that were added in 2007.

Details of the participants and the inclusion criteria are summarised in Table 1.

**Table 1:** Sample size and criteria for focus groups and interviews

Organisation	Focus groups	Individual interviews	Notes on selection process
CDFs	4 focus groups ranging from 8 to 11 participants from 4 to 6 villages. 35 people in all from 20 villages (3 did not send representatives)		1 or 2 participants from each CDF (one a long-term member; one with a shorter term).
Traditional Councils		2 (1) Traditional Council secretaries 2 Indunas	There are three Traditional Councils and one has only one village in the research site. Hence secretaries of only 2 Traditional Councils were interviewed. Each recommended an Induna for interview from within their Council, who had represented their village for the full 25 years of research.
Municipalities		2 (1) regional municipal managers 3 ward councilors	The PEO works with only 2 regional municipalities, and both regional municipal managers were interviewed. The PEO works with 9 ward councilors and interviews were done with the three who have the most villages in their wards included in the HDSS study area.
Department of Health	Focus group with managers of 8 Home Based Care Organisations (HBCs)	4 clinic managers	4 individual interviews with the clinic managers of the four busiest clinics and 1 focus group with the managers of all eight HBCs based at 8 clinics.
Department of Education		2 (1) education circuit managers	The PEO works with five education circuits, but only interviewed circuit managers from the two that have several schools in the research site.
Total participants	45 people in 5 FGDs	15 (11)	

*NB* Numbers in brackets represent final sample included in the paper

In total there were 60 participants, 45 of whom took part in the focus group discussions and 15 in individual interviewees. Unlike the local leaders, four of the service providers interviewed lived outside the site, so these were excluded from the analysis. They were a municipal manager, a traditional council secretary, a clinic manager and an education circuit manager. The final sample for analysis included individual interviews with 11 service providers rather than 15 and a total sample of 56 people. The participants were aged between 25 and 70 years, and were balanced by gender.

The questions in the topic guide for both the focus groups and the individual interviews focused on three topics: knowledge and understanding of the work of the research unit; experiences or involvement in the work of the unit; and perceived and real benefits and concerns regarding working with, and the work of, the unit. Probing questions were designed to explore participant’s knowledge on the focus of the research, and of current and completed research projects. Questions were also asked about whether research results were useful, if participants had ever been invited to events held by the unit, if they were involved in any other way in unit activities, or had been able to influence unit activities. The benefits of being involved in research as well as any concerns about involvement were also probed.

A limitation of the study is that interviews were conducted by the first author who is conversant in the local language and who manages the PEO office. She was aware of her positionality and steps were taken to mitigate any bias through having a local interpreter and note taker present at each interview. The researchers were aware of the possibility of participants giving socially desirable responses, and interviewees were encouraged to give honest responses even if that meant being critical.

### Analysis

All interviews and focus groups were recorded; with the exception of one where the participant consented to be interviewed but refused to be recorded so extensive field notes were written. The interviews were transcribed and translated from Shangaan into English where necessary. The interviews with the two indunas were conducted exclusively in Shangaan while in most other interviews a mixture of English and Shangaan was spoken. The transcripts were imported into the QSR NVivo software (version 10) [41] and analysed thematically both deductively and inductively [42]. The deductive approach was by coding the data according to the topics of the topic guide, and the inductive approach was the identification of emergent themes in the language of the respondents within the interviews such as ‘Keeping secrets’ or ‘Taking blood’. In order to deal with inter-rater reliability, two of the authors read a selection of the interviews independently to identify key themes which formed the initial coding template to which additional inductive codes were added.

## Results

**Fig. 2:**
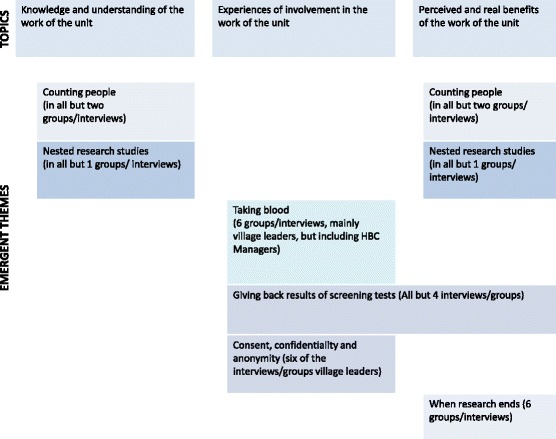
Themes emerging from topics, discussed by which participant groups/individuals

Themes that emerged during inductive analysis of the four topics, shown in Fig. 2, were: counting people, nested research projects, taking blood, giving back of screening test results, consent, confidentiality and anonymity, and when research ends. Counting of people was mentioned in all groups and interviews except for two, and nested research projects, both as an activity of the unit, as well as having some benefits to the unit were mentioned in all but one. Taking of blood was discussed in some detail in five groups/interviews, mainly by village leaders, but including the Home Based Care managers, mostly as a concern. Giving back results of screening tests created positive and negative comment, and was discussed in all but four interviews/groups. Consent, confidentiality and anonymity were again mostly a concern of village leaders i.e. discussed in six of the interviews/groups with the CDFs, ward councilors, traditional council secretaries and indunas. What happens when research ends was discussed in six groups/interviews, but was mostly of concern to the clinic operations managers.

### Counting people

The interviewees referred to the MRC/Wits-Agincourt Unit as ‘Wits’ which is the common shortened version of the University of the Witwatersrand used globally, and this term will be used throughout. When asked about the research activities in the area, the first activity that most participants mentioned was that Wits counts people, referring to the annual census of households.

*What I have noticed is that they are counting people. (Induna 2, Man)*

*Wits counts people. They also want to know how many people are employed in a household and how many people are living in that household. (HBC Manager FGD P2, Woman)*

Apart from general population variables (births, deaths, migrations), selected modules are added to the census at regular, albeit less frequent, intervals. For example food security is run every three years, and socio-economic status biannually until 2015 when it became annual. Not all participants understood the relevance of questions that were not purely demographic.

*There are complaints that the community have (I don’t know maybe it's true)… they say that sometimes it is frustrating when you come to their places and ask how do you eat? Meanwhile they have nothing on the plate. (Ward Councillor 2, Man)*

*We see Wits people going door to door asking questions like what you eat, about our toilets, cows, access to water, if we have a stove. I know this information might help you or assist the government to know about how people are living in their villages. But we as a community, how do we benefit? (CDF FGD 1, P4, Woman)*

*The questions about how often we eat meat are good because they want to know how vulnerable we are. (HBC Managers, FGD P4, Woman)*

The census is seen as providing useful information for planning of services for the Municipal Integrated Development Plan (IDP) and other service delivery planning such as the clinics knowing their catchment area statistics. Some participants were aware that results might not show immediate benefit at the local level, but would be useful at a national level. However, some participants were not fully aware of how results were, or could be used.

*In our village we looked at the results and found that our village is too small, so we went to the chief to extend our village by 500 new stands. (CDF FGD 2, P7, Man)*

*Every year we are getting the numbers of houses, numbers of young people, adults and numbers of those who have died, from Wits and it helps us because now we know our catchment area. (Clinic Manager 4, Woman)*

*Even though sometimes the research does not seem local, we know that sometimes it is important for the national and provincial levels’ (Clinic Manager 1, Woman)*

*Is there anywhere you send these research results - maybe the government is aware because you are taking something that you find and hand it over to the government to help with some problems that they are facing. (CDF FGD 1, P1, Man)*

Wits uses a different way of defining a household to that of the municipality, and this was mentioned as a problem when using HDSS statistics for planning. Households in publications are defined as those where members eat together, even if they sleep in buildings on a separate stand, whereas village leaders need to know how many actual occupied stands there are in order to provide services for each house.

*I can say I have used the results for profiling but Wits would say that there is one family if they all cook and eat in one place, but I suggest you expand that profiling to use stand numbers. Population is defined the same, but household is different. But I still use the data for development such as planning for water pipes and looking at indigent households. (Ward Councilor 2, Man)*

*Maybe my child she builds her own place to stay on a new stand, but when she wants to eat she comes to my place, but during the night she goes home to sleep. Wits counts her as living in my place. (CDF FGD 4, P1, Woman)*

### Nested research studies

Since 2000, the unit has had an increasing number of nested health research studies focusing on non-communicable diseases such as stroke and hypertension [43], HIV and other sexually transmitted diseases [8, 44], obesity and metabolic disease risk in children and adolescents [45, 46] and adult health and ageing [47, 48]. This has meant that members of the experimental public living within the HDSS area have been participants in different studies and as such have been part of smaller, shorter duration experimental publics, some of which have involved health screening. Each study is based on a sample drawn from the HDSS households and various screening tests have been administered to study participants at home and in the Wits research laboratory. Screening tests in different studies have included measurement of blood pressure, blood glucose, cholesterol, body mass index and HIV status and screening for stroke and epilepsy.

*In the past Wits was only counting people but now they are helping us as a community about different illnesses. (Induna 1, Man)*

*Wits helps us finding how many people are infected by illness and also helps us finding the number of people suffering from high blood pressure, sugar diabetes and the other illnesses like epilepsy. (CDF FGD 4, P2, Woman)*

Participants mentioned that there is an increasing awareness of symptoms, and the diseases with which they are linked, such as stroke, or heart disease. Some, like the clinic operational managers, believed that the results would assist them to improve service delivery as well as the health of their patients.

*Wits is helping us because people were hiding themselves without knowing the different illnesses that they were suffering from and were too afraid to go to the clinic. Now most people are aware of any illness they may have. (CDF FGD 3, P8, Woman)*

*The older people do not find it simple to attend clinics to check for high blood, sugar diabetes and everything, but Wits goes to the household directly and tests you for sugar diabetes and high blood and that’s helping very much. (FGD CDF 4, P4, Man)*

*I understand you are doing research, and the purpose of it I believe is to improve the health of patients because after your research, I understand you are going to bring some information here to help us……. If you carry on referring people with high blood to us, I think this can assist the patients. (Clinic Manager 2, Woman)*

People also mentioned how greater awareness of the health issues affecting people had encouraged some changes in behaviour at a village level.

*We now have a ladies soccer team, and when they gather they talk about HIV/AIDS and they are no longer scared about it. We also have the old age groups some of them are having high blood pressure and others TB so every morning we are going to the soccer field and we also have a machine to measure their blood pressure. (CDF FGD 2, P1 Female)*

### Taking blood and giving back results of screening tests

Not all biomarker results are given to research participants at the time of sample collection. In a population-based HIV prevalence study, participants were given a card with a study identifier barcode, and were asked to go to the clinic to collect their HIV result in two weeks [44] as the study team did not include trained HIV counsellors. This created problems for the clinic staff including not being able to find test results so having to turn participants away, and difficulties for lay counsellors who were not involved in the pre-test counselling but then had to handle the post-test counselling. This was not a method that study participants or clinic staff felt was the best way to deliver results.

*There was a project where patients had an HIV test in the community and they were given the bar code to come with to the clinic. Our lay counsellors were having a problem because when they had to counsel the person they had not done the pre-test counselling, but they had to give results - not an easy thing. (Clinic Manager 2, Female)*

*Let say sometimes people are coming to your household to do some tests - they don’t come and tell you that what we have found - they say you have to go to the office to find out. But they visited you to find out what is happening exactly with your life, so I think it should be advisable for them to come back to you as an individual and tell you the blood test results. (CDF FGD 4, P1, Woman)*

This raises an important ethics of practice issue concerning the way in which results from screening tests conducted in research studies are handled. While being screened at home was seen as beneficial, taking blood samples has raised some concerns. Some participants reported that villagers did not understand why blood was being taken or thought that blood was being sold by Wits.

*Those who are taken to Wits offices to have blood taken are saying that we are no longer going there because Wits is making money with our blood. (Induna 2, Man)*

*Lots of older people are running because people who went to Wits to give blood, they came back and started telling people that Wits takes blood and sells it. (CDF FGD 2, P5, Man)*

*I have never come across any challenges about the work of Wits besides those who are complaining about blood - that they don’t know what Wits wants do with their blood at the end. (Induna 1, Man)*

Concerns about blood taking are common in health research in Africa and are often framed in ways that illuminate relations between researchers and participants and social inequalities [25, 33, 35]. This recurring narrative of blood being sold, reveals an understanding that the collection of blood is integral to the scientific and economic enterprise of funded health research, although remains somewhat of a mystery.

### Consent, confidentiality and anonymity

Some of the local leaders reported that research participants sometimes run away or hide when they see Wits vehicles approaching, and some of the interviewees felt this was due to fear of being screened. Clearly, not being at home when fieldworkers visit is an indirect way of refusing consent to participate in a study. In this site, there are very low refusal rates for the annual census update (18 household refusals out of 21,500 households (0,1%) in 2016), and an average refusal rate of 5–10% for nested research studies. Studies drawing blood, particularly those with several tubes being collected, attract the highest refusal rates. The right to withhold consent is explained before every interview, but historically in this context, there is a difficulty in directly refusing participation as has been shown elsewhere in rural Sub-Saharan Africa [31]. Male village and municipal leaders who were interviewed presented themselves as possible advocates to use their influence to assist Wits in encouraging people to be research participants. These could be seen as misplaced good intentions in that informed consent needs to be given freely without duress or coercion.

*We as community leaders can contribute in enhancing awareness, particularly in those areas that Wits is facing challenges. (Municipal Manager 2, Man)*

*We can motivate our communities that they must give Wits people time when they arrive in their households, and they must also listen to them when they explain about their work and we can also encourage them to participate in their research. You know there are people who sometimes refuse to go to Wits when they want to take their blood because they are saying that Wits benefits from their blood. I know if they are being encouraged by us as community leaders, they will understand and they will participate easily. (Induna 2, Man)*

Another theme raised in the interviews was that of the importance of ‘keeping secrets’. Although there were some comments that Wits had a good reputation for keeping confidentiality, such as one FGD participant saying “Wits keeps confidentiality” (P6, Man, CDF FGD 3), there were worries expressed about secrets about their lives being published.

*“Yes, people run away when they see Wits cars because people don’t want to be published. Let’s keep the secrets.” (P6, Man)*

*“What I have experienced is that Wits comes to know everything about this particular person, and so that’s why people don’t want to be published. But you can ask me anything - I can give you answers so long as you keep my secret.” (CDF FGD 1, P3, Woman)*

There is an understanding amongst local leaders that Wits research involves not only collecting data but also publishing it, and a concern regarding confidentiality and anonymity in relation to the publication of research findings concerning aspects of their lives.

However, there were also requests from some representatives for research data that would identify for them individuals with problems, such as specific illnesses or extreme economic vulnerability, in order to help them. While leaders understand and value the need for anonymity and confidentiality, some would like confidentiality to be breached where the information can help individuals. In some cases, just being given epidemiological patterns and profiles was not regarded as sufficient.

*Is it possible for Wits to have an open debate about results that they get from households, or is it a secret thing if someone has got disease of some kind? We get basic information like ‘So many people have got this disease in your village’ but we don’t know who are those people. How can we then help those households in order to prevent such things, because Wits gets information and puts it in a secret, secret place? (CDF FGD 4, P7, Man)*

*We have a problem with people who don’t have identity documents and would like Wits to come find out who does and doesn’t have their documents, because we need to know how to help people whose parents were not born in South Africa and are now dead. (HBC Managers FGD, P3, Woman)*

Some of these concerns about confidentiality were directly related to doubts about trusting the local fieldworkers to keep secrets [24].

*I think some challenges my colleagues have already mentioned is the issue of privacy. Some Wits fieldworkers are just young people like us, and we meet them anywhere and a few of them could talk about my information in the wrong place at the wrong time. (CDF FGD 2, Man)*

*Remind the fieldworkers that they must keep confidentiality, when I give them the information they must keep it secret. (CDF FGD 1, Woman, P4)*

### When research ends

A trial on hypertension management [43] placed lay health workers in the clinics for two years, to assist with making appointments, calling patients to remind them of their appointments, completing the triage when patients arrived at the clinics (blood pressure, temperature and weight) and pre-packing medications. The clinic managers felt that the lay health workers assisted in increasing adherence to medication for people with hypertension, and were disappointed that the study ended, pointing out that the study had created some dependence on the lay health workers.

*People were benefiting a lot because those lay health workers kept reminding their clients about the date when they were supposed to collect their treatments. They also followed up those who were defaulting. (Traditional Council Secretary 2, Man)*

*It’s more painful to the clients because every day the patients are just complaining, and some of them are defaulting from the treatment because they are expecting the call. (Clinic Manager 4, Woman)*

The benefits of studies and effects of withdrawal from the field was also discussed in relation to a trial of a cash incentive to keep young women in secondary school in order to examine the impact on HIV incidence [8]. Data collection for this study took place at the weekends with transport provided to take groups of young women to the laboratory.

*They were taking the young girls during the weekend to teach them but since they are no longer taking them, the community is getting worried about why they are no longer taking them because they have noticed that it was keeping them busy. (CDF FGD 2, P1, Woman)*

## Discussion

This paper explores the views of local leaders and service providers who form part of the experimental public of a long running HDSS site on aspects of the ethics of practice [19]. Montgomery and Pool (2017) have set out a cogent argument for the replacement of the term ‘trial communities’ with that of ‘experimental publics’. Here, it is being applied to the setting of a longitudinal HDSS site which carries out an annual census of all households with additional studies including health service interventions, trials and observational studies. The qualitative data for this paper were from interviews with a sample of 56 leaders and service providers living in the 23 villages that had been part of the site since 2007, 19 of which have been involved since its inception in 1992.

The results suggest that this experimental public, which has been involved in an annual population census since 1992 and smaller time-limited studies since 2000, has developed a nuanced understanding of research activities, data collected for publication, and the relevance of results for policy especially locally, but also nationally and internationally.

The views and concerns of this experimental public highlight issues relevant to the ethics of practice of research, or ethics in the field, in HDSS and other longitudinal health research sites [19]. Fair benefit, both during and at the end of studies is an essential component of research planning and implementation [11, 13, 14, 26]. This is also one of more recent ethical considerations mentioned in the fifth revision of the Helsinki Declaration [27]. Our participants expressed a range of views on benefits, including need for individual benefits while acknowledging the value of government use of results for service planning, and appreciating that questions about food security shed light on vulnerability and inequality within rural populations. Village leaders and service providers also recognized that research results were useful for service planning in their areas.

Consent, confidentiality and anonymity, while considered an integral part of the process of procedural ethics (autonomy and the informed consent process [49]), are also important considerations for the ethics of practice. Societal norms impact on these processes [18, 28–30], an example being ‘silent refusals’ [31]. In this setting, there are high levels of consent but leaders and service providers reported that people sometimes hide when they see fieldworkers coming to their house. Indirect refusals are an indication of resistance in a setting where direct refusal may involve defying collective patriarchal consent negotiated at the village or household level. Another dimension of this was the view expressed by village leaders that they could use their influence to encourage recalcitrant individuals to take part in studies. In line with international guidelines [26], consent for the census and each study is first negotiated at the village level through meetings or letters and then at the individual level. These statements by the village leaders showed that they felt involved with giving consent at the village level but are not fully cognizant of individuals’ rights to refuse to participate in research.

‘Keeping secrets’ emerged as an issue expressed in a number of different ways by the interviewees. There were concerns that material published needed to keep the secrets of the research participants which relates directly to issues of confidentiality and anonymity. Small area, geographically defined HDSS sites can be identified in publications even though villages and individuals are anonymized [22]. In addition, local fieldworkers collect data, which they could link to individuals were they to talk about findings in public places – this was an expressed concern of the participants. On the other hand, some of the leaders and service providers expressed a wish for more personalized information as well as the aggregated data in order to respond to need at the village level which would breach confidentiality and anonymity [26]. This shows the complexity and sensitivity of anonymity and confidentiality issues within a longitudinal experimental public.

The collection of blood and other biological samples in research studies is a contested issue that causes concerns amongst experimental publics, in Africa as well as elsewhere [33, 36]. In Kenya researchers were characterised as ‘kachinga’, blood thieves [33], and in South Africa there were rumours that the researchers were selling blood for cash in a trial of Microbicide gels [25]. In this setting, there were reported concerns that blood taken during studies was being collected for sale. While not factually true, it captures the financing structure of international research in which biomarkers of the physical bodies of the experimental public sustain the scientific research activities and global reputation of a research site [50]. Dismissal of these concerns by researchers, who interpret them simply as rumours or distorted understandings, has often occurred, but anthropologists are clear that they need to be understood and contextualised historically, socially and culturally [34].

There were mixed views about the collection of blood and other screening tests. The participants felt these increased awareness of illness conditions resulting in people being more willing to seek health care. Also expressed, was a request for HIV screening results to be given to individuals at home rather than having to collect them at the clinic.

Another concern in guidelines on research in developing countries [10, 26] is what actions should be taken when the research ends. In this setting, the interviewees expressed concern about the withdrawal of a health service intervention introduced in a trial to improve screening and treatment of hypertension [43]. The lay health workers stopped their activities at the end of the two year study and both the service providers and village leaders commented on their withdrawal. Resources and manpower are scarce and therefore sustainability of health service interventions is rarely possible even if they are effective [51, 52]. Efforts at ensuring collaboration between researchers, health service providers and policy makers are becoming more mainstream in low income countries, but uptake of research results into future policy and practice may be limited owing to limited resources [38].

## Conclusion

There is a long history of international medical and public health research in Africa much of which is funded by international bodies based in the global north and carried out with participation of researchers from these countries. Recently there has been increasing focus by mainly social scientists and ethicists on key aspects of these activities including consent, anonymity and fair benefit. This paper makes a contribution to the emerging debate on the publics of public health research in Africa [1] and to the landmark research on aspects of ethics of practice and public engagement in longitudinal health research sites in sub-Saharan Africa [16, 20, 53]. In particular, this paper develops the concept of experimental publics proposed by Montgomery and Pool [36] to those within longitudinal HDSS sites, through exploring the views of local leaders and service providers within one established study setting. The concerns explored here on ethics of practice illustrate the complex dimensions of consent, anonymity, confidentiality and fair benefit which have implications for policy, practice and governance for those engaged in global health research in longitudinal health research sites internationally.

## Abbreviations

CDF: Community Development Forum
FGD: Focus group discussion
GLH: Gillian LewandoHundt
HBC: Home based carers
HDSS: Health and Socio-Demographic Surveillance System
HIV: Human Immunodeficiency Virus
HREC: Human Research Ethics Committee
IDP: Integrated Development Plan
IPHTRE: Improving Population Health Through Research Exchange
KK: Kathleen Kahn
MRC: Medical Research Council
PEO: Public Engagement Office/rs
RT: Rhian Twine
